# The perceived meaning of a (w)holistic view among general practitioners and district nurses in Swedish primary care: a qualitative study

**DOI:** 10.1186/1471-2296-8-8

**Published:** 2007-03-08

**Authors:** Eva Lena Strandberg, Ingvar Ovhed, Lars Borgquist, Susan Wilhelmsson

**Affiliations:** 1 Lund University, Department of Clinical Sciences, Malmö, Family Medicine, Malmö University Hospital, SE-205 02, Malmö, Sweden; 2 Blekinge County Council, SE-371 81 Karlskrona, Sweden; 3 General Practice, Department of Health and Society, Faculty of Health Science Linköping University, SE-581 83 Linköping, Sweden; 4 R&D Department of Local Health Care, County Council of Östergötland, SE-581 85 Linköping, Sweden

## Abstract

**Background:**

The definition of primary care varies between countries. Swedish primary care has developed from a philosophic viewpoint based on quality, accessibility, continuity, co-operation and a holistic view. The meaning of holism in international literature differs between medicine and nursing. The question is, if the difference is due to different educational traditions. Due to the uncertainties in defining holism and a holistic view we wished to study, in depth, how holism is perceived by doctors and nurses in their clinical work. Thus, the aim was to explore the perceived meaning of a holistic view among general practitioners (GPs) and district nurses (DNs).

**Methods:**

Seven focus group interviews with a purposive sample of 22 GPs and 20 nurses working in primary care in two Swedish county councils were conducted. The interviews were transcribed verbatim and analysed using qualitative content analysis.

**Results:**

The analysis resulted in three categories, attitude, knowledge, and circumstances, with two, two and four subcategories respectively. A professional attitude involves recognising the whole person; not only fragments of a person with a disease. Factual knowledge is acquired through special training and long professional experience. Tacit knowledge is about feelings and social competence. Circumstances can either be barriers or facilitators. A holistic view is a strong motivator and as such it is a facilitator. The way primary care is organised can be either a barrier or a facilitator and could influence the use of a holistic approach. Defined geographical districts and care teams facilitate a holistic view with house calls being essential, particularly for nurses. In preventive work and palliative care, a holistic view was stated to be specifically important. Consultations and communication with the patient were seen as important tools.

**Conclusion:**

'Holistic view' is multidimensional, well implemented and very much alive among both GPs and DNs. The word holistic should really be spelt 'wholistic' to avoid confusion with complementary and alternative medicine. It was obvious that our participants were able to verbalise the meaning of a 'wholistic' view through narratives about their clinical, every day work. The possibility to implement a 'wholistic' perspective in their work with patients offers a strong motivation for GPs and DNs.

## Background

The definition of primary care varies between countries. In a WHO report primary care was considered to be more difficult to define in comparison with specialist care, which is described as services delivered by specialists in hospitals [1]. In the same report 92 definitions of primary care were identified as originating from the United States. In European countries the definitions varied. Primary care can, according to the same report, be described in terms of concept, level, content of services, process and team membership.

Starfield describes primary care by level and function [2]. Primary care is according to Starfield the first level of care, which in turn provides an entry into the health care system for all health needs and problems, provides person-focused care over time, and coordinates or integrates care provided elsewhere or by others [2]. Starfield also defines primary care as a set of functions that, in combination, are unique to primary care and characterised by four attributes; first contact, longitudinality, comprehensiveness and coordination [2].

In Sweden, primary care has developed from a philosophic viewpoint and was first defined in the 1980s and is based on the concepts of quality, accessibility, continuity, co-operation and a holistic view. The definition of a holistic view was that all aspects of people's needs, psychological, physical and social, should be taken into account and seen as a whole [3].

Goldstein et al. meant that 'holistic health' and 'holistic medicine' were concepts frequently used to describe primary care in the United States [4]. However, the concept was not precisely defined and the meaning was unclear. In paragraph six in the WHO charter for General Practice/Family Medicine in Europe 'holistic' means the physical, psychological, and social perspective of individuals, families, and communities [5].

The word 'holism' or 'holistic view' is not a MeSH-term in the PubMed database while 'holistic health' and 'holistic nursing' were defined as follows [6]:

"Health as viewed from the perspective that humans and other organisms function as complete, integrated units rather than as aggregates of separate parts"

"A philosophy of nursing practice that takes into account total patient care, considering the physical, emotional, social, economic, and spiritual needs of patients, their response to their illnesses, and the effect of illness on patients' abilities to meet self-care needs"

It would appear that the meaning of holism in international literature differentiates between medicine and nursing. Medicine implies complementary and alternative medicine, while holistic nursing means to view all a patients' aspects in the present situation [7,8]. In their study from 1987 Goldstein et al. investigated differences between Holistic Physicians (HP) (members of the American Holistic Medical Association) and Family Physicians (FP). They pointed out that it was not obvious how to define holistic medicine. However, the results showed that HP had more spiritual and/or religious beliefs and use more holistic techniques compared to FP [4].

Primary care in Sweden is based on the five prestigious headings; quality, accessibility, continuity, co-operation and a holistic view. Apparently, of these headings the concept of a holistic view is the one least studied and described. The literature offers different meanings of the concept. Is then the difference between medicine and nursing due to different educational traditions? In medical training, over 80 percent of the content derives from biology and natural science, while in the nursing programme about 25 percent of the content derives from natural science and over 50 percent from behavioural science [9]. Olesen et al. suggest that it is time for general practice to revise the academic agenda and incorporate more influence taken from behavioural science into both the undergraduate curriculum and specialist training [10].

In Sweden, general practitioners (GPs) and district nurses (DNs) are the central actors in primary care. Primary health care is organised in primary care centres. In some centres GPs and DNs work separately and refer patients to each other and in other centres they work together in teams. Both GPs and DNs have responsibility for both preventive and medical/nursing care for all age groups.

Due to the uncertainties about holism and a holistic view, we wished to study, in depth, how they were perceived by doctors and nurses in their clinical work. Thus, the aim was to explore the perceived meaning of a holistic view among general practitioners and district nurses.

## Methods

We chose an inductive approach. Data was collected through focus group interviews. As a research technique the purpose is to collect qualitative data through interactional discussions with a specific focus [11]. The ideal group size is between 6 and 10 participants. Members of a focus group are selected from persons who may be expected to share their experiences of the phenomenon that would be of interest to the researcher. During the discussion, the participants are invited to communicate their ideas and experiences to each other, which will thereby encourage and stimulate further discussion thus more and more information will emerge. Together the group will form and develop answers to the researcher's questions. The interactive discussion will generate valuable details of complex experiences and reasons behind actions, beliefs, perceptions and attitudes [12].

### The participants

A purposive sample of 33 GPs and 26 DNs were invited to participate by letter. Eleven GPs and three nurses declined to take part in the interviews. Furthermore, two DNs did not participate, one due to workplace problems and the other because of illness. In total, seven focus group interviews were conducted. Four groups with altogether 22 GPs; 10 women and 12 men were interviewed (interviews 1 – 4). Three groups with in total 20 nurses, whereof 18 women and 2 men, participated (interviews 5 – 7). The interviews took place at care centres in two different county councils in the southern part of Sweden. When conducting focus groups it is important to ensure that the groups embrace a wide range of possible observations and therefore are made up of both men and women, of different ages, with different lengths of time in their professions and that urban as well as rural areas are represented in all groups. The data was collected from the Autumn of 2002 until Spring 2003.

Two of the authors, ELS and SW, conducted all the interviews together. The interviews took place at the R & D Units or in a primary health care centre, at the end of the working day.

A semi-structured interview guide was used. The interviews took from 90 to 120 minutes each. The focus of the discussion was 'what is a holistic view in primary care?'. After an opening presentation, each participant answered the question 'What does a holistic view in clinical every day life mean to me?'. Then a voluntary participant was requested to depict an episode, from their experience, characterised by a holistic view. This started a free association. The role of the moderator was to see that the participants did not deviate from the research question and that all participants had the opportunity to take part in the discussion. Another task for the moderator was to bring up the questions of the importance of having a holistic view and if having a holistic view is exclusive for primary care.

The interviews were audio taped and transcribed verbatim by a secretary. Additional notes by the assistant were added to the text of each interview.

#### Ethical considerations

Participation was voluntary. The collected data was coded and treated confidentially and is not linked to any named GP or nurse.

### Analysis

We found a latent content analysis, inspired by Berg and Graneheim & Lundman, to be the most suitable method for the purpose [13,14]. The analysis started with a naive reading of all the transcriptions of the authors independently. Subsequently an analysis took place by searching for units of meaning. Units related to the same content were gathered together and preliminary subcategories were developed. Subcategories were condensed into categories. To validate the categories a discussion among the four authors took place after an independent coding. To establish reliability the text was independently analysed by all four authors. Then the authors discussed the coding of units of meaning, subcategories, and categories until final agreement was reached. The findings were given to all of the participants. No one raised any objections to the developed categories and subcategories.

## Results

The content analysis resulted in three categories and eight subcategories (Table 1).

**Table 1:** Categories and subcategories from the content analysis

Category	Subcategory
Attitude	Professional attitude
	Political/administrative attitude
Knowledge	Factual knowledge
	Tacit knowledge
Circumstances	Motivating factor
	Organisation
	Sphere of activity
	Tool

### Attitude

One category was 'attitude' with the subcategory 'professional attitude' and 'political/administrative attitude'. Semantically the concept of a holistic view is not applied to Swedish primary health care other than in different policies and political documents, but was said to permeate GPs and nurses' practical work. It is more about ethical attitudes than tools and techniques, and as such it is significant for general practice (Table 2).

**Table 2:** Category 'attitude' and quotations of the subcategories 'professional attitude' and 'political/administrative attitude'

**Attitude**
*Professional attitude*
"In this connection a comprehensive view is an attitude that enables one to cover many dimensions in an illness or sickness, it's not necessary to have all the information – a comprehensive view does not mean that one has a lot more information" *(Interview 1)*
"I think it 's like a floating spirit that one has a kind of feeling about" *(Interview 4)*
"For me a comprehensive view is the opposite to a partial view, it's not just giving health care, it's all about not only being an ear specialist or not only being a psychiatrist or not only an orthopaedist and seeing only those problems but it is the comprehensive view" *(Interview 4)*
"We have like two conceptions, one including society and one about the whole person where you see not only the patient's wound but all other parts as well and that both body and soul in some way should feel all right. From there you make your professional decisions"*(Interview 7)*
*Political/administrative attitude*
"Yes, I must say that I have had a bit of a problem with this concept, a comprehensive view, because I have always thought of it as a prestigious word. I have never ever felt that I work with a comprehensive view, and when someone stands up and says that now we are going to work with a comprehensive view, then it has been a kind of, well bombastic talk from the director." *(Interview 2)*

#### Professional attitude

The participants discussed the concepts 'the whole' versus 'parts of the whole'. Many meant that the whole actually is greater than the sum of all the parts. It is all about finding the patient's hidden agenda and listening to what the patient is actually saying. Biomedical attitude is not enough. There is a need for a multidimensional viewpoint including a bio-psychosocial attitude towards the patients. GPs and nurses have to deal with the gap between 'illness' and 'disease', i.e. what the patient experiences and what is the medical problem. The need for both a medical overall picture and a holistic picture which include patients' social contexts as well as their body and soul, was strongly emphasised by the participants. To work from a holistic approach is all about the right ambitions for the individual patient 'here and now' (Table 2).

#### Political/administrative attitude

The concept 'holistic view' is politically heavy loaded and generally considered a rather empty phrase, seldom used by professionals for describing general practice. It is more of a prestigious guiding word taken from political documents (Table 2).

### Knowledge

The category 'knowledge' included the subcategories factual knowledge and tacit knowledge. All focus groups highlighted that a holistic view required knowledge including both theoretical knowledge and clinical practice (Table 3).

**Table 3:** Category 'knowledge' and quotations of the subcategories 'factual knowledge' and 'tacit knowledge'

**Knowledge**
*Factual knowledge*
"of course it is of great importance, we are skilled in it" *(Interview 5)*
"I don't think that we have so many regular meetings with physiotherapists and occupational therapists so it is not so important for me to get any extra information as most of it I have taken in throughout the years" *(Interview 1)*
*Tacit knowledge*
"without it there would be no primary care" *(Interview 1)*
"it's just there" *(Interview 5)*

#### Factual knowledge

Both GPs and nurses said that they had acquired their knowledge through their specialised training and throughout life long experience. GPs are skilled in natural sciences but compared to physicians working in hospitals the GPs said that they took many other things into account when meeting a patient, not only empirical knowledge (Table 3).

#### Tacit knowledge

The participants meant that a holistic view is not only factual knowledge but is also about feelings and social competence. Tacit knowledge expresses itself during the progress of working. It was also described by the participants as a metaphor of 'a floating spirit' and was said to be an essential prerequisite for primary care (Table 3).

### Circumstances

Both inner and outer circumstances can be barriers or facilitators for the possibilities to have a holistic view. The category 'circumstances' consists of four subcategories: motivating factor, organisation, sphere of activity and tools (Table 4).

**Table 4:** Category 'circumstances' and quotations of the subcategories 'motivating factor', 'organisation', 'sphere of activity' and 'tool'

**Circumstances**
*Motivating factor*
"It's just that which is the charm of the job" *(Interview 1,3)*
"It's that which makes everything else meaningful, to have a comprehensive view" *(Interview 7)*
*Organisation*
"and therefore I think that really having separate districts is good, if you want to see the entirety" *(Interview 6,7)*
"For me it is just when you come home to the elderly and so on that one sees not only the person, and they have been in contact with you earlier, but one sees the whole person, how they live" *(Interview 5,7)*
"I think that it is a little bit dangerous to say that we should specialise. GPs are not specialists. They have a general doctor's competence and I think that we as district nurses have a general nurse's competence or whatever we can call it, it is what we are trained as" *(Interview 6)*
*Sphere of activity*
"in preventive work I see a comprehensive view, how we can prevent more, it is in primary care where I see it, the comprehensive view" *(Interview 6)*
*Tool*
"There is the consultation, communication so to say, also a way to come to insight and to see for yourself . . . the comprehensive view of a situation, we need a tool really to draw this out" *(Interview 1)*

#### Motivating factor

All the focus groups, except one, brought up the fact that in primary care the possibility to act in accordance with a holistic view is a motivating factor in itself, being the core factor of general practice. It is essentially that what makes it fascinating and interesting to be a GP or a DN. 'It's that which makes everything else meaningful' (Table 4).

#### Organisation

The organisation of primary care affects the conditions for using a holistic view. A facilitating factor is the well-defined geographic districts, particularly for nurses. Teamwork is another factor of importance for understanding the patient's whole situation. The primary care team makes it possible to elucidate the patient's situation from different professional angles. Some participants noted that being the patient's GP for a long time might have the same impact and therefore the care team is then required to a less degree. To make house calls plays a central role for most of the participants particularly for the nurses but even for the GPs. Dividing primary care into different subcategories, for example special diabetic teams and high blood pressure surgeries, is possibly counter productive when it comes to achieving and maintaining a holistic view (Table 4).

#### Sphere of activity

Within certain spheres of activity the participants noted that it was particularly important, and obvious, to have a holistic view, for example in preventive work as well as in child health care. In palliative care, the nurses in particular said that it was important to be flexible and to be as 'a spider in the web' *(Interviews 5,6) *(Table 4).

#### Tool

The consultation and communication between patients and staff was considered important towards achieving a holistic view. Here there was a difference between the two professional groups. The GPs accentuated the consultation and communication with the patients more than the nurses. The nurses on the other hand emphasised the house calls more than the GPs (Table 4).

## Discussion

We found no uniform definition of the concept 'holistic view' in the literature. In the present study the content analysis revealed three different categories of the concept and the meaning and significance to the clinical life of Swedish GPs and DNs.

The concept 'holistic view' was first used in political documents, with the intention to characterise primary care in Sweden. In other descriptions and definitions, the concept of a holistic view seems to be implied in other concepts, for example in Starfield's definition or in the concept of personal care [2]. Continuity could be seen as one aspect of holistic care. Patients' perceptions were illuminated by Donahue et al. They found that patients were more satisfied with care given by physicians if they had had a longer period of seeing the same doctor. [15]. In another study Guthrie and Wyke found that it was especially important to have continuity of care if the patient had a chronic, multifaceted disease or emotional problems [16]. The results from these two studies correspond well with the findings from our focus group interviews, where especially the GPs discussed if continuity was necessary from a holistic viewpoint.

Tarrant et al. identified holistic care or 'whole person' care as a main feature of personal care among patients and providers in primary care settings in the UK [17]. In a recently published Swedish interview study, patients have expressed the importance of a holistic approach among GPs. This means that the doctor should be informed about a patients' whole life situation in order to be able to create a sense of security and coherence in the patient [18]. Particularly the nurses in the present study described themselves as specialists in this respect. Among the participants, the GPs put an emphasis on the consultation process as being an important tool for achieving a holistic view of the patients and their problems. Olesen et al has identified a number of important tools required to perform a multidimensional therapy as well as a balanced diagnosis and the consultation process is one such tool. Balanced diagnosis has to do with biomedical conditions, culture and context conditions, medico-psychological and social conditions. Another important tool is knowledge, theoretical and biomedical and also knowledge achieved by experience [19]. This relates well to our findings in both factual and tacit knowledge.

Both GPs and DNs mentioned the house call as being important in the process of achieving a holistic view of the patients and their special conditions. The nurses expressed the necessity of making the house calls themselves, whilst the GPs would rather acquaint themselves with nurses' experiences from their house calls. In many other countries GPs do house calls and in this aspect it is reasonable to believe that nurses' opinions could be transferred to GPs. When speaking of continuity and the individual patient, emphasis is on the integration and coordination of services. In this aspect nurses play a very important role for communication between nurses, but according to a Canadian study on delivery of acute care services in the home, the nurse practitioner also plays a central role both as a clinical expert and as case manager in order to integrate physician services in the home [20,21].

According to all the participants, how primary care is organised is important. The organisation can either facilitate or complicate the possibility to accomplish holistic and personal care. The primary care team, with its varied competence, will therefore facilitate a holistic view. Tarrant et al. expressed for example the importance of both the nurse and the receptionist together with the GP, in making care personal [17]. Also when talking about informational, management and relational continuity according to Haggerty et al. different professions have a role in linking information about the patient's preferences and context to ensure that the services are responsive to a single patient's needs [19].

There is always a risk of complications when translating complex concepts from one language to another. We have therefore made great efforts to find congruous terms. Ambiguities might also exist due to the difference in primary care systems in Sweden and in the UK and the US. The Swedish primary care system is based on organisational and administrative conditions with a publicly financed health care and a team based primary care, where district nurses have patients of their own and are independent co-workers to the GPs. In the UK and US systems, the nurses have a less independent role. We do not know whether these differences are reflected in the different languages and therefore influence the meaning of the concept. This deserves further study. The Swedish equivalents to 'comprehensive' and 'holistic' express similar differences as the English concepts. The Swedish district nurse is more concerned with primary care from a comprehensive viewpoint as 'the spider in the web', i.e. someone who considers the patients in their contexts, including their families and social circumstances. The statements from the GPs seem more holistic, i.e. more equal with general practice outside Sweden. The GP considers the patient in the context of the whole person, from biomedical, psychological and social perspectives [16,22].

There were few studies to be found that include the concept holistic view in the way we have defined it since it is not a MeSH-term. This meant that research on this topic might exist but was not retrievable. We found studies with different spellings of the concepts 'holism' and 'holistic'. Studies with the spelling 'wh' were more in alignment with our results, while studies with a simple 'h', were of a more philosophical or religious nature [4,23]. From the literature it would seem that holism is a subject that nurses have in their commission and not an assignment for GPs or other physicians, except in the case of very special groups of doctors such as the members of the American Holistic Medical Association [4].

However, in the present study it was obvious that GPs found a holistic view both meaningful and present in their daily work, only they did not name it as such. One could say that the concept was introduced by politicians and policy makers as a new brand of primary care, but was incorporated into Swedish primary care by the two main professional groups the GPs and DNs. Maybe a holistic view was already in place by the time of the introduction of the Swedish primary health care system and maybe all the policy makers did was to put a name on an already existing phenomenon. From the participants' narratives about their daily clinical work, it was evident that a holistic view characterised primary care as a whole. Their statements were distinguished by a homogeneous approach to the identity, the culture and the image of primary care, factors significant for an established brand [24]. We had an inductive approach when we asked our participants about their experiences. We were curious about what the concept 'holistic view' meant to the two main professional categories in Swedish primary care and we found a concept that was vividly alive in spite of the different theoretical backgrounds of the two professions: GPs' natural sciences against nurses' caring and behavioural sciences. The analysis of the interviews gave us a meaning of the concept. When we compared our findings with what is already known from the two research areas, nursing science and medicine, we found that spelling holistic with 'wh' instead of 'h' corresponded better to our participants' mutual understanding of the concept.

As the aim of this study was to explore the perceived meaning of a holistic view among GPs and DNs, a qualitative design was judged as relevant to gain a deeper understanding how the professions perceived the concept in their daily work, as the concept has not earlier been clearly defined. Focus group interviewing was deemed to be a suitable data collecting method. The purpose of focus group interviewing is to use group interaction to produce data. To prevent GPs or DNs from feeling uncomfortable and unable to speak freely in the group discussions we chose to interview the doctors and nurses separately. Focus groups are valid if they are used carefully for a problem suitable for focus group inquiry, and if they follow established procedures [11].

To do group interviewing needs training and the role of the moderator is to facilitate discussions between the group members. The two moderators (ELS, SW) have earlier conducted group interviews and were aware of the difficulties and were familiar with group processes and interviewing.

The findings from this study cannot be generalised but this is not the intention in qualitative studies, the main aim of which is to contribute to increased understanding. It is nevertheless reasonable to assume that our findings can be transferred to similar contexts.

## Conclusion

The concept 'holistic view' is multidimensional, well implemented and very much alive among both GPs and DNs. The word holistic should really be spelt 'wholistic' to avoid confusion with complementary and alternative medicine. It was obvious that our participants were able to verbalise the meaning of a 'wholistic' view through narratives about their clinical, every day work. The possibility to implement a 'wholistic' perspective in their work with patients offers a strong motivation for GPs and DNs 'Without a wholistic view there will be no primary care!' *(Interview 1)*.

## Competing interests

The authors declare that they have no competing interests.

## Authors' contributions

ELS and SW were involved in study design, conducted and analysed the interviews, and wrote the paper. ELS will serve as guarantor for the integrity of the data. IO and LB were both involved in the study design, analysed the interviews and made important contributions to the manuscript. All the authors read and approved the final manuscript.

## Pre-publication history

The pre-publication history for this paper can be accessed here:


